# Comprehensive Evaluation of the Volatomic Fingerprint of Saffron from Campania towards Its Authenticity and Quality

**DOI:** 10.3390/foods11030366

**Published:** 2022-01-27

**Authors:** Rosaria Cozzolino, Matteo Stocchero, Rosa Perestrelo, José S. Câmara

**Affiliations:** 1 National Research Council (CNR), Institute of Food Science, Via Roma 64, 83100 Avellino, Italy; 2 Department of Women’s and Children’s Health, University of Padova, 35122 Padova, Italy; matteo.stocchero@unipd.it; 3 Centro de Química da Madeira—CQM, Campus da Penteada, Universidade da Madeira, 9020-105 Funchal, Portugal; rmp@staff.uma.pt; 4 Departamento de Química, Faculdade de Ciências Exatas e da Engenharia, Campus da Penteada, Universidade da Madeira, 9020-105 Funchal, Portugal

**Keywords:** *Crocus sativus* L., volatile organic compounds, headspace solid-phase microextraction, gas chromatography mass spectrometry, multivariate statistical analysis

## Abstract

The volatile profiles of eight saffron samples (seven cultivated and one spontaneous) grown in different geographical districts within the Campania region (southern Italy) were compared. Using headspace solid-phase microextraction coupled to gas chromatography–mass spectrometry (HS-SPME/GC-MS), overall, 80 volatiles were identified in the eight landraces. Among them, safranal and its isomers and other related compounds such as isophorones, which are not only key odorants but also pharmacologically active metabolites, have been detected in all the investigated samples. Principal Component Analysis performed on the volatiles’ compounds revealed that the spontaneous sample turned out to be an outlier. In particular, the volatile organic compounds (VOCs) profile of the spontaneous saffron presented four lilac aldehydes and four lilac alcohol isomers, which, to the authors’ knowledge, have never been identified in the volatile signature of this spice. The multivariate statistical analysis allowed the discrimination of the seven cultivate saffron ecotypes in four well-separated clusters according to variety. Moreover, 20 VOCs, able to differentiate the clusters in terms of single volatile metabolite, were discovered. Altogether, these results could contribute to identifying possible volatile signature metabolites (biomarkers) or patterns that discriminate saffron samples grown in Campania region on a molecular basis, encouraging future biodiversity programs to preserve saffron landraces revealing valuable genetic resources.

## 1. Introduction

Saffron, the dried red stigma of flowers of *Crocus sativus* L., is highly valued for its unique aroma, taste and color. It is called “the red gold” and is currently considered to be the world’s most expensive spice [1,2]. Saffron has been known for four millennia and probably originated in the Middle East or in Greece (the southern Aegean islands of Crete and Santorini) [3]. Although it is principally used as a natural dye and as a flavoring in culinary preparations, several pharmacological properties, including antioxidant, anti-inflammatory, antidepressant and anticancer, have been confirmed for saffron [3]. In a very recent study, saffron has been also indicated as a promising anti-inflammatory and antiviral herbal medicine in the prevention of severe COVID-19 symptoms [4].

With a world production of 418 t per y-1, this spice can be cultivated in a range of different pedo-climatic conditions, which significantly impact its composition and quality. Iran is the principal producer with 90% of global production followed by India, Afghanistan, Morocco, and Euro-Mediterranean countries, including Greece, Spain and Italy [3]. In recent years, in Euro-Mediterranean basin countries there has been a drastic reduction in saffron production, mainly caused by highly manual, tedious and labor-intensive work requirements for stigma harvesting and separation and lack of modernization of the cultivation methods [3].

Freshly harvested saffron stigmas are almost odorless, as odoriferous substances appear during the drying and storage period developing the typical pleasant odor. The dehydration is the crucial post-harvest step toward obtaining saffron with suitable quality, since during drying, different physical, chemical and biochemical processes occur achieving the desired final properties of saffron, including aroma formation, chemical stability, and antimicrobial activity [2].

Saffron quality is determined by its three main secondary metabolites, crocin, picrocrocin and safranal, which are responsible for the color, bitter taste and odor, respectively [1]. Crocin is the main carotenoid of saffron which is formed after the glycosylation of crocetin, a dicarboxylic carotenoid. It is responsible for the intense color that saffron gives in aqueous solutions. Picrocrocin, a monoterpene glycoside, is the precursor of safranal and has only been detected in the *Crocus* genus, the only edible species of which is the C. *sativus*. Safranal, produced by the hydrolysis and dehydration of picrocrocin, is a volatile monoterpene aldehyde. This metabolite constitutes more than 65% of the total aroma components in saffron [1].

Saffron aroma comprises more than one hundred volatiles which can be mainly classified into (1) mono and sesquiterpenes, originated by the isoprenoid synthesis pathway; (2) phenylpropanoids and benzenoids produced in shikimic acid pathway; and (3) compounds derived from the enzymatic conversion of lipid peroxidation [2]. Safranal is the key component of saffron essential oil and can be measured by the absorbance of aqueous saffron extract at 330 nm (ISO 3632-1, Geneva, ISO 2003). This compound also exhibits antioxidant and cytotoxic properties against specific cancer cell lines [5]. However, the simple spectrophotometric method does not consider other volatile compounds, such as the isomers of safranal and isophorones, which not only deserve particular attention as key odorants in saffon, but also present therapeutic properties [2,5].

Geographical origin is considered a critical factor in the concentration of different volatiles detected in this spice. Traditional production of saffron complies with country-specific agronomic and post-harvesting techniques, so that C. sativus ecotypes display distinct qualitative grades of spice [3]. Specifically, Italy boasts some of the most valuable saffron in Europe with three Protected Designations of Origin (PDO): Sardinia, L’Aquila (Abruzzo) and San Gimignano (Tuscany), although there are also high-quality cultivations in Sicily and in the Campania region (South Italy). Many papers have been published on Italian saffron’s volatile organic compounds (VOCs) [2]. Nevertheless, to the best of our knowledge, no information is available on the volatile composition of saffron from the Campania region. Furthermore, in general saffron profiles of VOCs have not been comprehensively characterized, likely due to the complexity of the volatile fraction. Currently, the food-quality program of the European Union promotes food-origin protection through PDO and Protected Geographical Indication (PGI) to preserve biodiversity and ensure quality of the final product [6]. The increased interest for safranal and its related volatile compounds, as both aroma compounds and rewarding pharmacological agents, prompted us to undertake research on Campania saffron to determine simultaneously the amount of safranal and related volatile compounds. In particular, this study has been focused on the comparative characterization of the VOCs profiles of eight saffron samples grown in different geographical districts within the Campania region: three samples harvested in the province of Avellino, a sample cultivated in Benevento, a sample farmed in the province of Caserta, and three samples collected in the province of Naples.

The authenticity and typicity of samples, such as foods and spices, can be assessed through VOCs investigation. To this purpose, solvent-free headspace solid-phase microextraction (HS-SPME) followed by capillary gas chromatography–mass spectrometry (GC-MS) represents one of the most powerful analytical tools for the targeted or untargeted detection of VOCs. A plethora of studies has revealed that the volatile signature obtained by HS-SPME/GC-MS combined with chemometric tools could be used as an authenticity marker to define the variety and geographical origin, as well as to detect frauds for different foodstuffs, including saffron, providing local growers with numerous benefits [2,7]. In this context, comprehensive VOCs profiles of the abovementioned eight saffron samples have been obtained by HS-SPME/GC-MS method and the volatomic data were treated by multivariate statistical analysis, with the aim of contributing to the characterization of the volatile components and to identify possible signature metabolites (biomarkers) or patterns that discriminate the samples under examination.

## 2. Materials and Methods

### 2.1. Chemicals and Materials

All reagents used in this study were of analytical quality. Sodium chloride (NaCl, 99.5%) was supplied by Panreac (Barcelona, Spain), while 3-octanol and the C8–C20 n-alkanes mixture were purchased from Sigma-Aldrich (St. Louis, MO, USA). Ultrapure water (18 MΩ cm at 23 °C), obtained from a Milli-Q system (Millipore, Bedford, VA, USA), was used throughout. The SPME fibers (divinylbenzene/carboxen on polydimethylsiloxane-DBV/CAR/PDMS, with 50/30 μm film thickness and 1 cm fiber length), the SPME holder for manual sampling and the amber glass screw cap vials for SPME with PTFE/silica septa were from Supelco (Bellefonte, PA, USA). Helium (ultrapure grade, Air Liquide, Algés, Portugal) was used as the carrier gas in the GC system.

### 2.2. Saffron Samples

The stigmas of saffron (*Crocus sativus* L.) of the eight landraces, harvested in 2021, were provided by local growers from different geographical districts within the Campania region (southern Mediterranean area of Italy), through local committee promoters. In detail, three samples, including GB (Fontanarosa, 600 m a.s.l.; latitude: 41°01′05″ N; longitude: 15°01′13″ E), EC (Capriglia, 575 m a.s.l.; latitude: 40°57′40.68″ N; longitude: 14° 46′41.16″ E), AP (Lacedonia, 730 m a.s.l.; latitude: 41°3′7.92″ N; longitude: 15°25′28.92″ E), were from the province of Avellino, one sample, designated with BN, was cultivated in Benevento (400 m a.s.l.; latitude: 41°7′49,80” N; longitude: 14°47′13.20″ E), one sample, indicated as TL, was farmed in the province of Caserta (Raviscanina, 300 m a.s.l; latitude: 41°22′16.32″ N; longitude: 14°14′36.96″ E), and three samples, collected in the province of Naples, were labelled as MC (Ottaviano, 220 m a.s.l; latitude: 40°51′2.88″ N; longitude: 14°29′27.60″ E), RR, and RRWT (Agerola, 630 m a.s.l.; latitude: 40°38′19.32″ N; longitude: 14°32′22.92″ E). In particular, RRWT sample refers to a spontaneous ecotype.

Each saffron ecotype was grown, harvested and processed in agreement with the guidelines provided by the collective brand “Zafferano Italiano” (https://www.zafferanoitaliano.it (accessed on 1 December 2021)). Individual saffron samples, harvested at the end of the growth cycle, were dried at a temperature inferior to 45 °C with a final a moisture content not superior to 12%. All the saffron samples, stored in small glass jars, showed stigmas with a length ranging from 1 to 3.5 cm which in the extreme upper part had broad and cylindrical papillae and presented a purple–red color.

### 2.3. Volatile Organic Compounds (VOCs) Analysis

#### 2.3.1. Sample Preparation and HS-SPME Procedure

The optimization of HS-SPME experimental parameters was carried out by analyzing commercial saffron samples obtained from a local market (Madeira Island).

For each variety, saffron stigmas were ground into a fine powder just before the analysis. The sample preparation procedure was the following: for each replicate, 1 g of each powdered saffron sample was mixed into a 20 mL amber glass vial to 0.5 g of NaCl, 6 mL of ultra-pure Milli-Q water and 5 µL of 3-octanol (0.4 µg/mL), used as the internal standard (IS). Successively, a magnetic stirrer was added into the vial which was capped with a PTFE-faced silicone septum and positioned in a thermostatic bath at 45 ± 1 °C to equilibrate the system. The SPME was executed in headspace mode (HS), where the fiber was fixed to a manual SPME holder and exposed to the HS sample for 50 min under constant magnetic stirring (800 rpm). The VOCs extracted by SPME were thermally desorbed by the direct insertion of the fiber into GC injector at 250 °C for 10 min, in splitless mode.

SPME fibers were conditioned as suggested by the manufacturer, but below the maximum recommended temperature prior to their first use. Before the initial daily analyses, the fibers were conditioned at 250 °C for 10 min into the GC injector port and the blank level was checked. Experiments were performed in triplicate for all samples, except for RRWT saffron that was analyzed in duplicate.

#### 2.3.2. Gas Chromatography- Mass Spectrometry Analysis (GC-MS)

Chromatographic separations of volatile metabolites from the eight saffron ecotypes were performed using an Agilent 6890N (Agilent Technologies Palo Alto, CA, USA) gas chromatography system equipped with a SUPELCOWAX^®^10 fused silica capillary column (60 m × 0.25 mm i.d. × 0.25 μm film thickness) supplied by SGE (Darmstadt, Germany), and coupled to a mass spectrometer 5975 C (Agilent, Santa Clara, CA, USA) with a quadrupole inert mass selective detector (Santa Clara, CA, USA). The oven temperature program was initially set at 40 °C for 1 min and then increased to 220 °C at 2.5 °C/min and held for 10 min, for a total GC run time of 83 min. The flow of He, the carrier gas, was kept at 1 mL min^−1^, while the injection port operated in the splitless mode at 250 °C. For the VOCs detection, the operating temperatures of the transfer line, quadrupole, and ionization source were 270, 150, and 230 °C, respectively, while the electron impact (EI) mass spectra were recorded at 70 eV ionization voltages and the ionization current was 10 µA. The electron multiplier was set to the auto-tune mode and the mass acquisitions range was 30–300 *m*/*z*. The resulting chromatograms were processed by using the Enhanced ChemStation software for GC-MS (Agilent Technologies, Palo Alto, CA, USA).

Volatile metabolites were identified by matching their mass spectra with those reported in the standard NIST05/Wiley07 libraries, by comparing the retention indices (RI) (as Kovats indices), calculated relative to the RT of a series of n-alkanes (C8–C20) with linear interpolation, with of the data from the literature, and by comparison of the GC RT of the chromatographic peaks with those, when available, of pure standards run under the same conditions. For individual volatiles, the peak area was measured from the total ion chromatogram (TIC) and semi-quantified by relative comparison with the peak area of the IS (Relative Peak Area, RPA%).

### 2.4. Statistical Data Analysis

Exploratory data analysis was performed by Principal Component Analysis (PCA) [8]. PCA is an unsupervised multivariate technique able to summarize the data variation of a data table using a small set of score components, called principal components, calculated by linear combination of the measured features. The model of the data table obtained by PCA approximates the original data structure providing a less complex data-representation, where the similarity between observations and the correlation structure among the measured features are preserved. Moreover, since PCA discovers the structured data variation removing both random noise and redundant data variation due to multicollinearity, the space spanned by the principal components provides a more useful data representation for clustering than that obtained by the original features. Using the principal components as coordinates to represent the observations, Hierarchical Cluster Analysis (HCA) based on the Euclidean distance and the Ward’s method was applied, to discover relevant clusters of observations. The optimal number of clusters was determined by Silhouette analysis [9]. PCA was also used for outlier detection applying the Hotelling’s T2 and the Q tests.

The clusters discovered combining PCA and HCA were characterized in terms of single VOCs using the Kruskal–Wallis test controlling False Discovery Rate (FDR) by Benjamini–Hochberg procedure [10]. The distributions of the most interesting features were represented by boxplots.

The relationships between VOCs and altitude were explored by correlation analysis. Specifically, Spearman’s correlation coefficient was calculated for individual volatile and altitude. Controlling FDR by Benjamini–Hochberg procedure, a set of VOCs related to altitude was discovered and the results were represented using the Volcano plot.

Data analysis was performed by in house R-function implemented, using the R 4.0.4 platform (R Foundation for Statistical Computing, Vienna, Austria).

## 3. Results and Discussion

### 3.1. Volatomic Profile of Saffron Samples

The volatile profiles obtained from the eight saffron samples analyzed are quite different (Figure S1).

**Figure 1:**
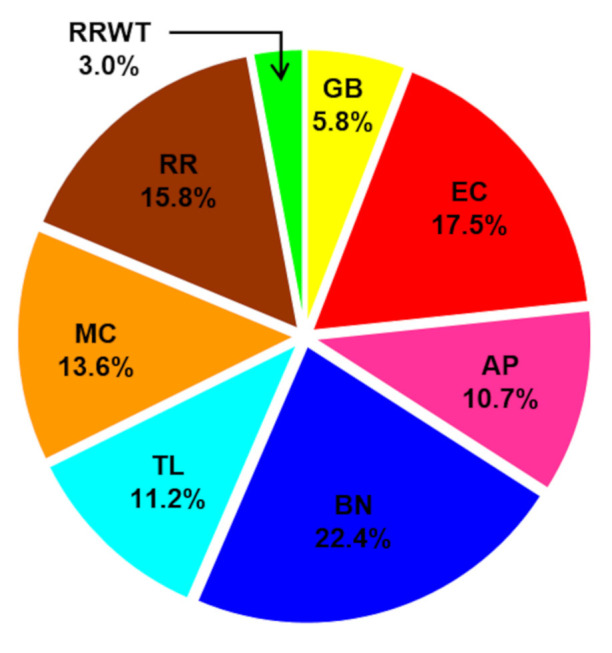
Profile of total volatile fraction in the saffron samples from the different geographical sites: GB—Fontanarosa; EC—Capriglia; AP—Lacedonia; BN—Benevento; TL—Caserta (Raviscanina); MC—Ottaviano; RR—Agerola; and RRWT—Agerola (spontaneous ecotype).

**Figure 2:**
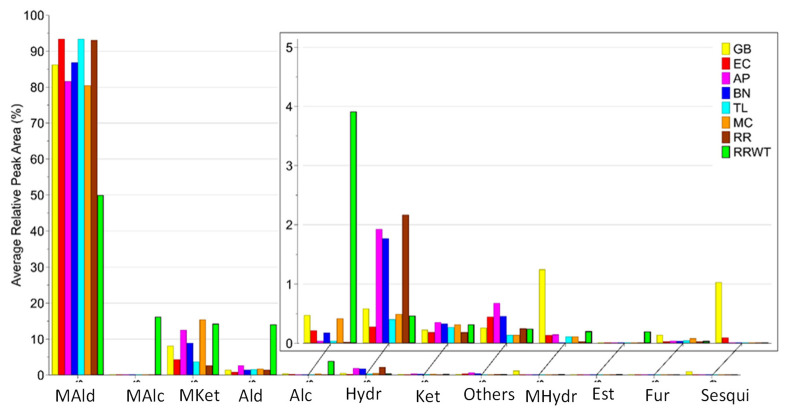
Distribution of VOCs identified in the eight saffron samples from the different geographical sites, by chemical family: MAld—monoterpene aldehydes; MAlc—monoterpene alcohols; MKet—monoterpene Ketones; Ald—aldehydes; Alc—alcohols; Hydr—hydrocarbons; Ket—ketones; MHydr—monoterpene hydrocarbons; Est—esters; Fur—furans; Sesqui—sesquiterpenoids.

Overall, 80 volatiles were identified by HS-SPME/GC-MS analysis in the eight saffron landraces belonging to the following chemical classes: monoterpene ketones (12), monoterpene hydrocarbons (10), monoterpene aldehydes (7), monoterpene alcohols (6), aldehydes (14), alcohols (7), ketones (7), hydrocarbon (7), furans (3), sesquiterpenes (2), esters (2), and others (3). The identified 80 VOCs, the abbreviation code, the experimental and literature Kovats index and the identification methods are reported in Table 1. For each identified VOC, the medians of the RPA% values and the corresponding range are reported in Table S1. Moreover, Figure 1 reports the volatile content detected in the saffron samples from different geographical sites, while Figure 2 indicates that monoterpene aldehydes, followed by monoterpene ketones, are clearly the most abundant VOCs identified in all the saffron ecotypes.

Among the 80 compounds detected, most of them have been already reported in saffron from diverse geographical origins and obtained by various extraction and detection techniques [2,5,11,12,13,14,15,16,17,18,19]. Seventeen VOCs were common to all the eight ecotypes, including hexanal (V12), heptanal (V18), limonene (V19), 2-pentylfuran (V21), nonanal (V37), 1,3,5,5-tetramethyl-1,3-cyclohexadiene (V38), β-isophorone (V39), benzaldehyde (V46), α-isophorone (V53), safranal (V57), 4-ketoisophorone (V59), 2,4-dimethylbenzaldehyde (V65), dihydrooxophorone (V69), nepetalactone (V74) 2-(butylthio) thiophene (V78), and 4-(3-methyl-2-butenyl)-4-cyclopentene-1,3-dione (V79). Consistently with literature data, terpenes were the most abundant class, both qualitatively and quantitatively, with monoterpenic aldehydes and ketones being the most represented group [5]. Safranal is the principal constituent of all the saffron samples, ranging from 92% in EC, TL and RR to 43% in RRWT of the total VOCs content, in line with almost all reports (Table S1) [2]. Safranal is a monoterpenic aldehyde (2,6,6-trimethyl-1,3-cyclohexadiene-1-carboxaldehyde) which is nearly absent in the fresh stigma of saffron [18]. It is synthesized from the bitter glycoside picrocrocin by an enzymatic process and/or during the drying and the storage phases, either by generating the intermediate 4-hydroxy-2,6,6-trimethyl-1-cyclohexen-1-carboxaldehyde (HTCC), which is converted by dehydration to safranal, or directly by thermal degradation [1,2,20].

Saffron minor volatiles can be classified relying on their chemical structures and/or precursors [2,18]. The first group consists of compounds with structures that bear a distinct similarity to that of safranal, also reported as isophorone analogs (C9 and C10 groups of compounds). Among them, β-isophorone (V39), phorone (V47), α-isophorone (V53), 2-hydroxyisophorone (V58), 4-ketoisophorone (V59), and dihydrooxophorone (V69) have been identified in almost all the investigated samples of the present study (Table S1).

The second group of minor aromatic molecules of saffron refers to constituents with a partially unsaturated C4 chain in the 1-position. They are generated from the degradation of lipophilic carotenoids during the stigmas’ maturation and includes the ionone derivatives [18,21]. This group, detected in saffron regardless of its geographical origin, plays a crucial role in the synthesis of new compounds during the storage process and has been indicated as key aroma compounds, responsible for the herbaceous and floral notes in saffron [2,5,11,12,14,17,18]. In all the examined samples, excepting the RRWT ecotype, compounds of this group, including 6-methyl-5-heptene-2-one (V34), α-ionone (V53), dihydro-β-ionone (V70), trans-geranyl acetone (V71), β-ionone (V75), and dihydro-β-ionol (V77), were detected (Table S1). The third group of minor volatile constituents are the saturated linear hydrocarbons [2].

Among the C10 volatile components, besides saffron and its isomers, β-cyclocitral (V55) was present in the volatile patter of EC, TL and MC samples, while HTCC (V80) has been only found in the cultivated samples (Table S1). β-cyclocitral, also present in other plants, has been described as an important component of flavor and aroma in many fruits, vegetables and ornamental plants contributing in attracting pollinators [5,16].

The RRWT sample showed 12 VOCs that were not detected in the cultivate ecotypes (V25, V30, V36, V45, V48, V49, V50, V51, V60, V62, V63, and V68), while 10 VOCs (V22, V31, V32, V33, V34, V35, V70, V71, V75, V80) found in the cultivated samples were not observed in the spontaneous saffron (Table S1).

Among the volatiles exclusively found in the RRWT sample, the compounds V49, V50, V51, and V52 correspond to the lilac aldehyde isomers A, B, C, and D, respectively, while the components V60, V62, V63, and V68 refer to the lilac alcohol isomers A, B, C, and D, respectively (Table S1). These oxygenated monoterpenoids are from the most complicated chiral floral scent compounds and have been identified among the main volatile components in many plants, most of all ornamental species, harvested all over the world, as important metabolites for the attraction of pollinators. In addition, these VOCs have been also described as key volatile markers in several honeys [22,23,24]. To the authors knowledge, lilac aldehyde and lilac alcohol isomers have never been detected in the VOCs profile of saffron.

### 3.2. Discrimination of Saffron Samples

**Figure 3:**
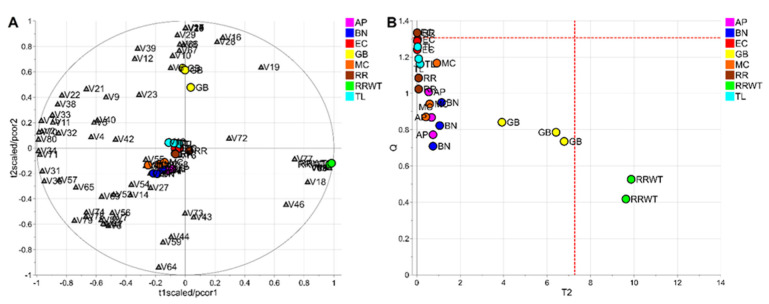
PCA model of the whole data set: biplot (**A**) and T2/Q plot used to detect outliers (**B**); dashed red lines indicate the limits at the level of confidence of 95%.

To evaluate the potential of the volatile profiles obtained by HS-SPME/GC-MS in the discrimination of saffron samples according to the variety, a statistical analysis of the volatomic data matrix was accomplished. Specifically, the data set composed of 23 observations (3 technical replicates for each variety except for 2 replicates for the spontaneous variety, RRWT) and 80 VOCs was investigated by PCA. Data were log-transformed and auto scaled before performing data analysis. The model showed two principal components with R_2_ = 0.65 and the relative biplot is reported in Figure 3A.

The two observations of the RRWT sample are located at the limit of the plot, suggesting a relevant difference with respect to the other observations (Figure 3A). Indeed, assuming α = 0.05, the replicates of the spontaneous sample were outliers, as it is possible to observe in Figure 3B, where the T2/Q plot is illustrated.

**Figure 4:**
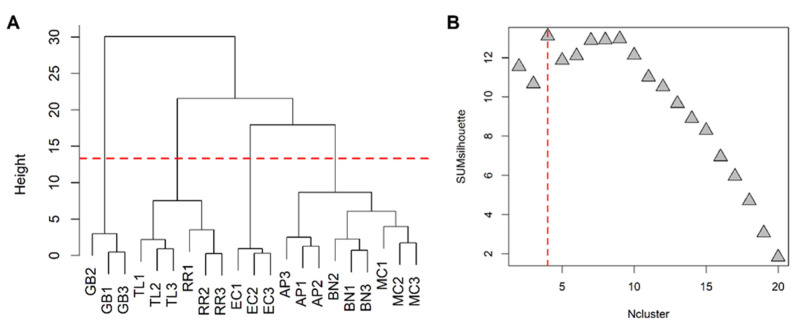
HCA performed on the space spanned by the the principal components of the PCA model built excluding the wild-type samples: dendrogram (**A**) and Silhouette plot (**B**); SUMsilhouette is the sum of the silhouette calculated for each single observation and Ncluster is the number of clusters. Since the maximum of SUMsilhouette is obtained for Ncluster = 4, the optimal number of clusters was 4.

**Figure 5:**
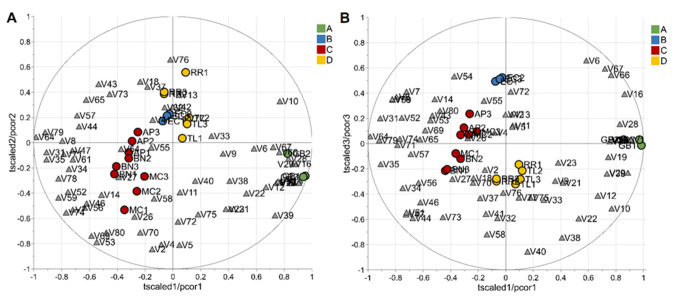
Biplots (**A**,**B**) of the PCA model built excluding the wild type variety; observations are colored according to cluster membership; grey triangles indicate the VOCs. The first and the second principal component explained 37% and 17% of the total variance, respectively, while the total variance explained by the third component was 15%. Letters A, B, C, and D refer to the four clusters identified by HCA, and their composition, in terms of saffron variety, is reported in Table 2.

**Figure 6:**
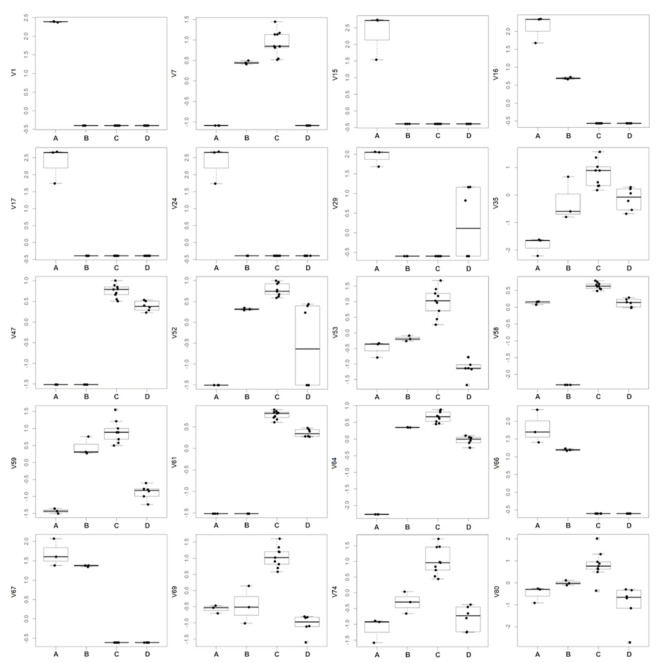
Boxplots of the VOCs selected by Kruskal-Wallis test controlling FDR by Benjamini–Hochberg procedure (δ = 0.01).

Excluding the RRWT sample, the data set composed of the remaining 21 observations were log-transformed and autoscaled and investigated by PCA. Considering a model with three principal components and R^2^ = 0.69, HCA allowed us to identify four clusters of observations. In Figure 4 the dendrogram and the Silhouette plot used to define the optimal number of clusters is reported. It is worth noting that the data variation associated to the technical replicates is less relevant than that associated to the biological variety, as technical replicates of the same saffron sample are clustered together. Table 2 lists the composition of the identified clusters in terms of the saffron ecotype, while the biplots of the PCA model, where the observations are coloured according to the cluster membership, are described in Figure 5, which showed that the four clusters are in different regions of the plots and are well-separated. By Kruskal–Wallis test controlling FDR at the level δ = 0.01, 20 VOCs, able to characterise the clusters in terms of single volatile metabolite, were discovered (the *p*-values of the tests are reported in Table S2 of Supplementary Materials). In Figure 6, the boxplots of the selected VOCs are reported.

Compared to the other samples, in the cluster A, corresponding to the GB sample, 8 VOCs, including dimethyl sulphide (V1), β-myrcene (V15), α-phellandrene (V16), α-terpinene (V17), γ-terpinene (V24), *trans*-β-terpinolene (V29), β-sesquiphellandrene (V66) and α-curcumene (V67) showed the highest amount, while 1,2,3-trimethylbenzene (V35), and 4-ketoisophorone (V59), were the less abundant components. Moreover, GB is the only sample in which eucarvone (V64) has not been detected (Table 2; Figure 6; Table S1). In particular, V1, V15, V17, and V24 have been found only in the GB sample (Table 1). To our knowledge, dimethyl sulphide (V1) and β-sesquiphellandrene have been identified for the first time in saffron. β-Myrcene (V15), described with a peppery odor, has been reported in Greek saffron stigmas dried by the traditional method [11], α-phellandrene (V16), characterized by a citrus, slightly green, black pepper-like odor, has been detected in the headspace analysis of a French saffron sample by using both GC×GC-ToF-MS and GC-ToF-MS [18], while α-terpinene (V17), γ-terpinene (V24), and terpinolene (V29), all having a citrus and herbaceous smell, have been first identified by Condurso et al., among the volatile constituents of Sicilian (South Italy) saffron samples [5]. Moreover, V24 has been recently detected in the volatile profile of a raw Spanish saffron sample [25]. Finally, α-curcumene (V67) has been found in the VOCs profiles of both stigmas and petals of Iranian saffron ecotypes with different geographical provenances and cultivated under different nutritional regimes [17]. Terpenes (mono and sesquiterpenes) are the most prevalent constituents in the essential oils of numerous plants, including saffron, and many of these metabolites have recognized potential biological effects. Specifically, V15, V16, V17, V24, and V29 and have been reported to exhibit antibacterial, antifungal, antiproliferative, antitumor, analgesic, and anti-inflammatory activity [26,27]. Several studies have also demonstrated that the combination of different monoterpenes produce a remarkable synergistic effect enhancing their biological properties [28].

Cluster B, comprising only the EC sample, is characterized by the lowest amount of 2-hydroxyisophorone (V58), an isophorone-related metabolite. The highest content of V58 was detected in the AP, BN, and MC samples which are grouped in cluster C (Table 2; Figure 6; Table S1). Specifically, with respect to the other ecotypes, samples of cluster C, besides V58, presented the highest concentration of the other 11 volatiles, including 2,5-dimethylfuran (V7), 1,2,3-trimethylbenzene (V35), phorone (V47), 6-methyl-3,5-heptadiene-2-one (V52); α-isophorone (V53), 4-ketoisophorone (V59), *trans*-acetaldehyde, (3,3-dimethylcyclohexylidene) (V61), eucarvone (V64), dihydrooxophorone (V69), nepetalactone (V74), and 4-hydroxy-2,6,6-trimethyl-1-cyclohexen-1-carboxaldehyde (HTCC) (V80) (Table 2; Figure 6; Table S1). Specifically, V47, V53, V58, V59, V64, and V69 are isophorone-related compounds (C_9_ and C_10_ group of compounds) which seem to be formed through the oxidation and decarboxylation of saffranal, but it has been suggested that they could also be generated through enzymatic pathways [11]. These volatiles, with a chemical structure similar to that of safranal, have been identified, by different analytical methods, as the most dominant metabolites in the volatile profile of saffron stigmas harvested in different pedo-climatic conditions and from different geographical areas, including several Italian regions [5,11,12,13,16,17,18,19,20,29,30]. The amount of these safranal-related volatiles has been reported to increase with drying temperature and preservation time of the saffron, being detected in higher amounts in 1-year dried samples respect to the freshly dried spice, conferring, together to safranal, the characteristic spicy and floral notes of saffron [16,20,29,30]. Prolonged storage over 3 years causes the degradation of these metabolites with a consequent increasing of caramel, citrus, and vegetable notes [2]. Very recently, these VOCs have been reported as freshness markers, demonstrating that the ratio of ketoisophorone to safranal can be used as an aging indicator [2].

Based on the screening of numerous saffron samples, V80 is among the major compounds of the VOCs profile of this spice and has been detected as an aroma-active compound [13,31]. Several studies conducted on saffron samples of different geographical provenance have described that during a 1 to 4 years’ storage period the amount of safranal remained mostly constant, while the quantity of HTCC reduced along the storage time [11,30]. Finally, the compounds V7, V35, and V52 have been previously detected in the stigmas and flowers of several Iranian saffron samples, while the ketone V52 has also been identified in Turkish saffron after one year of storage [17,30].

Cluster D, composed of the RR and TL samples, presented the lowest amount of α-isophorone (V53) and 4-ketoisophorone (V59) which have been discussed above as being among the most abundant volatiles in samples of cluster C (Table 2; Figure 6; Table S1).

Finally, controlling FDR at the level δ = 0.05, 7 VOCs, including 2-methylbutanal (V4), 3-methylbutanal (V5), 4-ethyl resorcinol (V14), 6-methylheptan-2-one (V22), 3-octanone (V26), 6-methyl-5-hepten-2-one (V34), and β-cyclocitral (V55), reported in Table S3 were found to be inversely correlated to the altitude, as meters a.s.l. of the cultivation site of the investigated saffron samples.

## 4. Conclusions

In this study, HS-SPME/GC-MS was used to characterize, for the first time, the volatile profile of eight saffron samples (seven cultivated and one spontaneous) grown in different geographical districts within the Campania region (southern Italy). Overall, 80 volatiles were identified in the eight landraces. Among them, safranal, its isomers, and isophorone-related compounds were reported to be not only key odorants but also responsible for the pharmacological activity showed by saffron extracts, and have been detected in all the investigated samples.

PCA performed on the volatiles compounds showed that the spontaneous saffron (RRWT) was an outlier. Particularly, the VOCs profile of RRWT presented four lilac aldehyde and four lilac alcohol isomers, which, to the authors’ knowledge, have never been detected in the volatomic fingerprint of this spice. Multivariate statistical analysis allowed the discrimination of the seven cultivate saffron samples in four distinct clusters according to variety. Moreover, 20 VOCs, able to differentiate the clusters in terms of single volatile metabolite, were discovered. Noticeably, the clear definition of these biomarkers would require dedicated studies, extending both the number of samples and the biological replicates.

Overall, our findings could promote future breeding programs aimed at safeguarding and preserving the production of the investigated saffron landraces from the Campania region despite the presence of biologically active volatile constituents characterized by relevant health and sensory properties.

## Figures and Tables

**Table 1:** Volatile metabolites detected in saffron samples and their identification code.

Metabolite	Code	^a^ RI_cal_/RI_lit_	^b^ ID	Metabolite	Code	RIt/RIsp	ID
**Furans**							
2-Methylfuran	V2	857/858	RI/MS/S	1-Butanol	V13	1144/1144	RI/MS/S
2,5-Dimethylfuran	V7	949/949	RI/MS/S	4-Ethylresorcinol	V14	1151	MS/S
2-Pentylfuran	V21	1241/1241	RI/MS/S	1-Pentanol	V23	1252/1253	RI/MS/S
**Esters**				1-Hexanol	V36	1357/1357	RI/MS/S
Ethyl acetate	V3	871/871	RI/MS/S	1-Octen-3-ol	V41	1455/1455	RI/MS/S
Methyl heptanoate	V30	1297/1299	RI/MS/S	Benzeneethanol	V72	1936/1937	RI/MS/S
**Aldehydes**				**Ketones**			
2-Methylbutanal	V4	903/903	RI/MS/S	1-Penten-3-one	V10	1025/1025	RI/MS/S
3-Methylbutanal	V5	907/907	RI/MS/S	6-Methylheptan-2-one	V22	1248/1247	RI/MS
Pentanal	V9	984/984	RI/MS	3-Octanone	V26	1265/1265	RI/MS/S
Hexanal	V12	1084/1084	RI/MS/S	6-Methyl-5-hepten-2-one	V34	1350/1351	RI/MS/S
Heptanal	V18	1195/1195	RI/MS/S	Ethanone, 1-(1,4-dimethyl-3-cyclohexen-1-yl)	V43	1484/1491	RI/MS
Octanal	V32	1301/1301	RI/MS/S	3,5-Octadien-2-one	V45	1538/1536	RI/MS
cis-2-Heptenal	V33	1340/1339	RI/MS	6-Methyl-3,5-heptadiene-2-one	V52	1611/1587	RI/MS
Nonanal	V37	1406/1406	RI/MS/S	**Hydrocarbons**			
trans-2-Octenal	V40	1448/1451	RI/MS/S	5-(1,1-dimethylethyl)−1,3-cyclopentadiene	V8	966	MS
Benzaldehyde	V46	1551/1553	RI/MS/S	Toluene	V11	1046/1046	RI/MS/S
4-Methylbenzaldehyde	V56	1655/1655	RI/MS	Styrene	V27	1274/1274	RI/MS/S
Trans-Acetaldehyde, (3,3-dimethylcyclohexylidene)	V61	1757/1799	RI/MS	Mesitylene	V31	1299/1297	RI/MS/S
2,4-Dimethylbenzaldehyde	V65	1771/1742	RI/MS	1,2,3-Trimethylbenzene	V35	1357/1355	RI/MS/S
2,4,6-trimethylbenzaldehyde	V73	1936/1929	RI/MS	1,3,5,5-tetramethyl-1,3-Cyclohexadiene	V38	1415/1406	RI/MS
**Alcohols**				Benzene, 1-methoxy-2-(1-methylethenyl)-	V44	1520	MS
Ethyl alcohol	V6	921/921	RI/MS/S				
**Monoterpene hydrocarbons**							
β-Myrcene	V15	1166/1166	RI/MS/S	Eucarvone	V64	1765/1756	RI/MS
α-Phellandrene	V16	1177/1171	RI/MS/S	Dihydrooxophorone	V69	1839/1839	RI/MS
α-Terpinene	V17	1190/1190	RI/MS/S	dihydro-β-ionone	V70	1857/1854	RI/MS/S
D-Limonene	V19	1209/1209	RI/MS/S	trans Geranyl Acetone	V71	1871/1870	RI/MS/S
γ-Terpinene	V24	1257/1257	RI/MS/S	Nepetalactone	V74	1947/1915	RI/MS
β-Ocimene	V25	1262/1262	RI/MS	trans-β-Ionone	V75	1967/1964	RI/MS/S
p-Cymene	V28	1285/1287	RI/MS/S	4-Cyclopentene-1,3-dione, 4-(3-methyl-2-butenyl)-	V79	1999/-	MS
Terpinolene	V29	1291/1291	RI/MS/S	**Monoterpene aldehydes**			
Megastigma-7(E),9,13-triene	V42	1466/-	MS	Lilac aldehyde A	V48	1561/1550	RI/MS
Megastigma-4,6(E),8(E)-triene	V54	1643/1568	RI/MS	Lilac aldehyde B	V49	1574/1565	RI/MS
**Monoterpene alcohols**				Lilac aldehyde C	V50	1583/1573	RI/MS
Eucalyptol	V20	1219/1219	RI/MS/S	Lilac aldehyde D	V51	1606/1597	RI/MS
Lilac alcohol isomer A	V60	1741/1736	RI/MS	β-Cyclocitral	V55	1649/1638	RI/MS/S
Lilac alcohol isomer B	V62	1759/1756	RI/MS	Safranal	V57	1673/1648	RI/MS/S
Lilac alcohol isomer C	V63	1764/1763	RI/MS	4-Hydroxy-2,6,6-trimethyl-1-cyclohexen-1-carboxaldehyde (HTCC)	V80	2159/2152	RI/MS
Lilac alcohol isomer D	V68	1806/1800	RI/MS	**Sesquiterpenes**			
Dihydro-β-ionol	V77	1978/1977	RI/MS	β-Sesquiphellandrene	V66	1789/1783	RI/MS
**Monoterpene ketones**				α-Curcumene	V67	1791/1791	RI/MS
β-Isophorone	V39	1428/1429	RI/MS/S	**Others**			
Phorone	V47	1556/1565	RI/MS	Dimethyl sulfide	V1	732/733	RI/MS/S
α-Isophorone	V53	1623/1621	RI/MS	Heptanoic acid	V76	1972/1972	RI/MS/S
2-Hydroxyisophorone	V58	1687/1675	RI/MS/S	2-(Butylthio)thiophene	V78	1996/-	MS
4-Ketoisophorone	V59	1719/1717	RI/MS/S				

^a^ RI_calc_: experimental Kovat’s index. RI_lit_: Kovat’s index reported in the literature. ^b^ Identification method as indicated by the following: RI—Kovats retention index on a on HP-Innowax column; MS—NIST and Wiley libraries spectra; S—co-injection with authentic standard compounds, where commercially available, on the HP-Innowax column.

**Table 2:** Clusters identified by HCA and their composition in terms of saffron variety.

Cluster	Variety
A	GB
B	EC
C	AP,BN,MC
D	RR,TL

## Data Availability

Excluded statement.

## Supplementary Materials

The following are available online at https://www.mdpi.com/article/10.3390/foods11030366/s1, Figure S1: Representative chromatograms of the eight saffron samples (see text for the sample code). Numbers above the peaks refer to the VOCs identified in Table 1. (1) β-Isophorone; (2) Benzaldehyde; (3) α-Isophorone; (4) Safranal; (5) 2-Hydroxyisophorone; (6) 4-Ketoisophorone; (7) Lilac aldehyde isomer A, (8) Lilac aldehyde isomer B; (9) Lilac aldehyde isomer C; (10) Lilac aldehyde isomer D; (11) Lilac alcohol isomer A; (12) Lilac alcohol isomer C; (13) Lilac alcohol isomer D. Table S1: VOCs concentration expressed as median [min–max] in the investigated samples. Table S2: VOCs selected by Kruskal–Wallis test controlling FDR by Benjamini-Hochberg procedure (δ = 0.01); *p* is the *p*-value of the Kruskal–Wallis test. Table S3: Spearman’s correlation coefficient *ρ* and its *p*-value (*p*) for the VOCs selected by FDR (δ = 0.05).
