# Supplementary material for: Comprehensive Evaluation of the Volatomic Fingerprint of Saffron from Campania towards Its Authenticity and Quality
Source: Foods. 2022 Jan 27;11(3):366. doi: 10.3390/foods11030366 (PMC8834390; doi:10.3390/foods11030366)
Supplement: Supplementary file 1 [file foods-11-00366-s001.zip › foods-1551538-supplementary.pdf]

Comprehensive evaluation of the volatomic fingerprint of Saffron from Campania region towards its authentication and quality control

Rosaria Cozzolino [1], Matteo Stocchero [2], and José S. Câmara[3,]

SUPPLEMENTARY MATERIAL

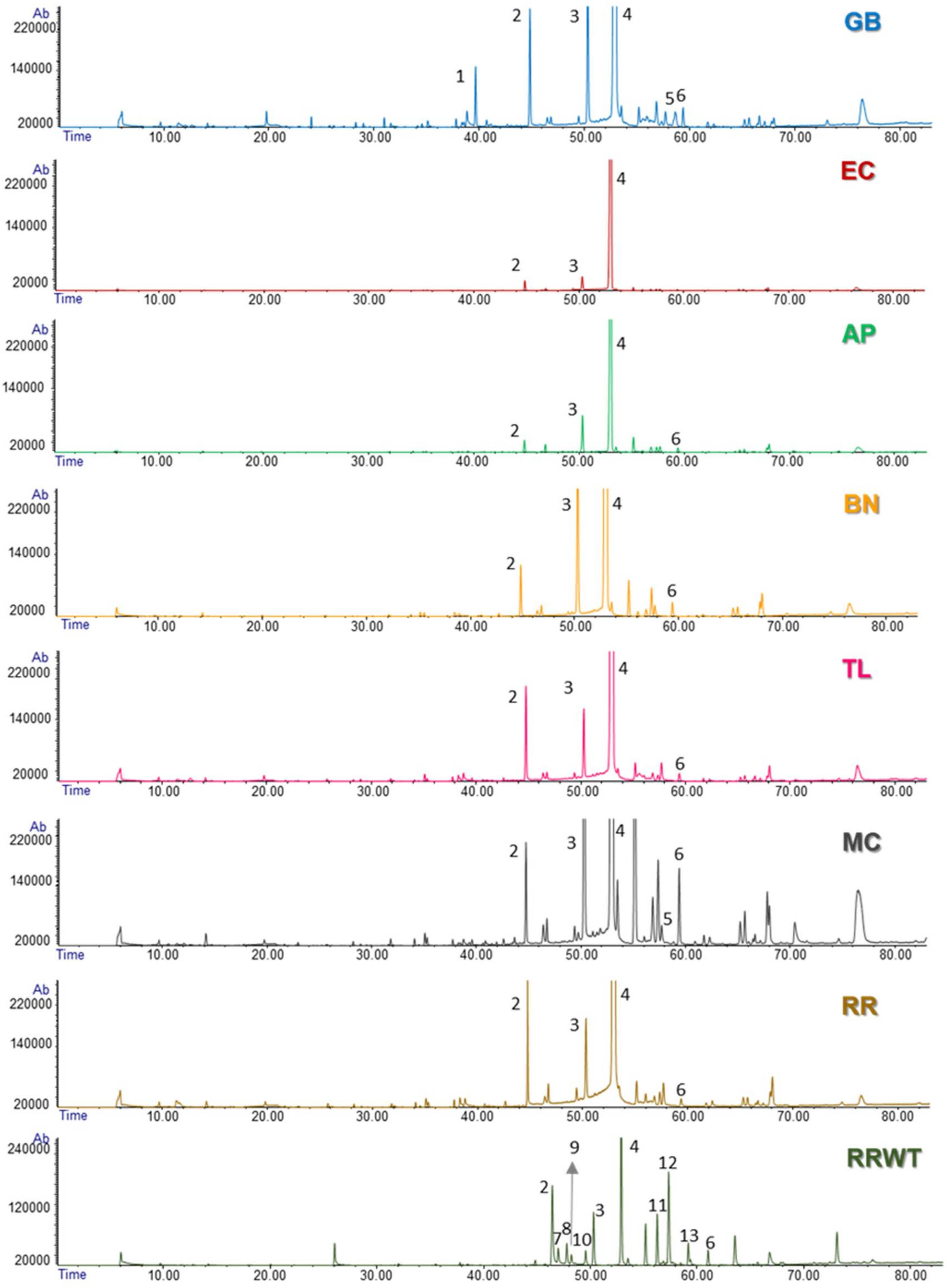

Figure S1. Representative chromatograms of the eight saffron samples (see text for the sample code). Numbers above the peaks refer to the VOCs identified in Table 1. (1) β-Isophorone; (2) Benzaldehyde; (3) α-Isophorone; (4) Safranal; (5) 2-Hydroxyisophorone; (6) 4-Ketoisophorone; (7) Lilac aldehyde isomer A, (8) Lilac aldehyde isomer B; (9) Lilac aldehyde isomer C; (10) Lilac aldehyde isomer D; (11) Lilac alcohol isomer A; (12) Lilac alcohol isomer C; (13) Lilac alcohol isomer D.

Table S1. VOCs concentration expressed as median [min-max] in the investigated samples.

ID[a]	GB	EC	AP	BN	TL	MC	RR	RRWT
V1	31.3 [30.6-31.7]	0 [0-0]	0 [0-0]	0 [0-0]	0 [0-0]	0 [0-0]	0 [0-0]	0 [0-0]
V2	31.7 [27.6-32.4]	20.3 [17.7-20.6]	22.9 [18.1-37]	34.4 [27.8-42.2]	24.1 [17.8-38]	54.4 [44.2-68.1]	18.9 [18.9-22.8]	0 [0-0]
V3	17.7 [0-18.1]	0 [0-0]	0 [0-0]	0 [0-0]	0 [0-0]	0 [0-0]	0 [0-0]	0 [0-0]
V4	35.6 [17.8-36.4]	11 [10.5-12]	27.5 [17.6-33.7]	62.2 [45.5-100.8]	36.8 [26.3-39.7]	47.8 [44.4-59.7]	0 [0-0]	0 [0-0]
V5	24 [23.5-42.3]	11.9 [11.3-12.9]	8.4 [8.3-13.2]	42.5 [29.4-47.9]	22.2 [13.6-30.8]	37.2 [23-40.3]	0 [0-0]	0 [0-0]
V6	221.6 [64.2-226.6]	53.1 [50.5-57.8]	32.3 [0-110.8]	0 [0-0]	0 [0-0]	0 [0-0]	0 [0-0]	0 [0-0]
V7	0 [0-0]	6.5 [6.2-7]	7.6 [7.3-11.2]	11.8 [11.7-17.6]	0 [0-0]	18.6 [17.6-27.1]	0 [0-0]	0 [0-0]
V8	0 [0-0]	239.4 [156.9-411.1]	206.4 [179.5-298.8]	270.5 [162.4-280.1]	0 [0-0]	254.4 [202-273.6]	97.1 [84.9-150]	0 [0-0]
V9	32.6 [24.3-33.3]	15.1 [14.4-16.5]	0 [0-0]	21.8 [15.2-34]	23.8 [16.3-23.9]	25.9 [21.1-27.6]	19.1 [18.9-32.6]	0 [0-0]
V10	28.4 [15-29.1]	0 [0-0]	0 [0-0]	0 [0-0]	12 [8.3-13.6]	0 [0-0]	16.2 [12.9-18.3]	0 [0-0]
V11	61 [20.9-62.4]	23 [21.8-25]	64.8 [56.7-86.8]	26.3 [25.2-45.4]	18.9 [12.6-26.7]	22.9 [20.8-30.1]	27.3 [11.1-28.6]	0 [0-0]
V12	267.2 [198.5-286.4]	77.5 [42.1-100.7]	65.1 [53.3-76.7]	105.9 [95.2-137.2]	173.8 [145.2-198.2]	78.3 [63.3-127]	71.6 [56.4-110.6]	49.9 [42.1-57.7]
V13	0 [0-0]	8.4 [8-9.2]	0 [0-0]	0 [0-0]	12.1 [11.2-12.9]	0 [0-0]	0 [0-0]	0 [0-0]
V14	0 [0-0]	36.5 [34.7-39.8]	0 [0-0]	65.2 [59-74.7]	0 [0-0]	44.6 [43.3-53.4]	0 [0-0]	0 [0-0]
V15	30.8 [7.5-31.5]	0 [0-0]	0 [0-0]	0 [0-0]	0 [0-0]	0 [0-0]	0 [0-0]	0 [0-0]
V16	501.2 [120.5-512.3]	13.7 [13-14.9]	0 [0-0]	0 [0-0]	0 [0-0]	0 [0-0]	0 [0-0]	6.9 [5.5-8.2]
V17	19.3 [7.3-19.7]	0 [0-0]	0 [0-0]	0 [0-0]	0 [0-0]	0 [0-0]	0 [0-0]	0 [0-0]
V18	36.9 [36.1-39.1]	83.2 [79.1-90.6]	34.6 [29.9-38.9]	128.4 [106.2-142.1]	84.4 [79.4-89.7]	32.5 [21-39.8]	85.5 [85.1-91.4]	1055.7 [931.9-1179.4]
V19	70.2 [21.3-71.7]	13.4 [12.8-14.6]	7.2 [7.1-12.7]	17.2 [16.4-19]	12.1 [9.9-15.1]	10.6 [8.8-16.1]	12.8 [12.2-17.1]	42.4 [39.9-44.9]
V20	94.7 [18.5-96.8]	0 [0-0]	0 [0-0]	0 [0-0]	0 [0-0]	0 [0-0]	0 [0-0]	0 [0-0]
V21	86.2 [58.5-88.2]	37.1 [35.2-40.4]	22.3 [20.1-29.9]	54.3 [51.5-59.8]	38.1 [37.8-49]	73.7 [58.7-76.5]	45.2 [42.4-58.4]	14.1 [14-14.2]
V22	53.2 [49.3-54.4]	11.5 [11-12.5]	12.2 [11-18.5]	25.6 [23.4-27.6]	61.7 [55.1-64.4]	29.4 [24.8-35.5]	18.1 [15.7-19.7]	0 [0-0]
V23	14.6 [12.3-14.9]	0 [0-0]	7.4 [2.8-10.5]	0 [0-0]	13.8 [13-14.4]	12.9 [10.4-20.2]	0 [0-0]	0 [0-0]
V24	15.2 [6-15.5]	0 [0-0]	0 [0-0]	0 [0-0]	0 [0-0]	0 [0-0]	0 [0-0]	0 [0-0]
V25	0 [0-0]	0 [0-0]	0 [0-0]	0 [0-0]	0 [0-0]	0 [0-0]	0 [0-0]	12.7 [11.9-13.6]
V26	0 [0-0]	0 [0-0]	0 [0-0]	0 [0-0]	0 [0-0]	12.8 [10-19.2]	0 [0-0]	0 [0-0]
V27	0 [0-0]	0 [0-0]	0 [0-0]	49.4 [33.2-56.3]	0 [0-0]	0 [0-0]	0 [0-0]	0 [0-0]
V28	472.2 [116.8-482.8]	17.8 [16.9-19.4]	0 [0-0]	0 [0-0]	12.2 [0-13.3]	12.6 [6.5-18.5]	10.2 [5.3-14.5]	15.1 [14.8-15.5]
V29	91.3 [48.5-93.3]	0 [0-0]	0 [0-0]	0 [0-0]	19.3 [10.4-19.4]	0 [0-0]	0 [0-0]	0 [0-0]
V30	0 [0-0]	0 [0-0]	0 [0-0]	0 [0-0]	0 [0-0]	0 [0-0]	0 [0-0]	75.1 [63.5-86.7]
V31	37.2 [19.8-38]	134.3 [105.4-169.8]	169.8 [140.6-176.1]	151.7 [149.9-175.8]	72.8 [70.9-102.8]	121.8 [99.6-148.5]	89.4 [53-94]	0 [0-0]
V32	27 [13.7-27.6]	25.1 [23.8-27.3]	11.6 [7.8-25.7]	61.8 [35.8-65.4]	49.5 [30.3-59.1]	14.9 [11.8-20.7]	50.5 [32.3-66.1]	0 [0-0]
V33	48.3 [16.7-68.2]	23.6 [22.4-25.7]	18.1 [14.8-24.8]	32.9 [20.4-36.9]	29.1 [19.3-39.8]	23 [19.6-45.1]	45.5 [35.3-55]	0 [0-0]
V34	100.4 [74.6-111.3]	171 [162.5-186.2]	141.9 [123-166.9]	340 [298.4-392.7]	233.8 [197.9-260.2]	247.3 [225.3-323.5]	186.5 [147.2-193.8]	0 [0-0]
V35	30.6 [19.7-31.3]	69.4 [59.4-181]	155.2 [141.5-214.7]	306.8 [237.8-359.1]	92.9 [64.9-114.6]	140.7 [125-215.1]	129.3 [72.4-135.6]	0 [0-0]
V36	0 [0-0]	0 [0-0]	0 [0-0]	0 [0-0]	0 [0-0]	0 [0-0]	0 [0-0]	72.1 [62.8-81.5]
V37	93 [69.9-95.1]	144.8 [137.6-157.7]	65 [52.7-78.7]	301.1 [249-310.6]	158 [112.2-198.9]	55.9 [51.1-88.3]	301.9 [151.8-465.6]	77.7 [77.4-78]
V38	333.5 [326.8-334.1]	107.2 [101.9-116.7]	141.6 [119.4-147.3]	306.9 [305-332.1]	303.9 [255.2-328.1]	149.2 [138.4-221.9]	201 [170.8-206.8]	13.5 [13.2-13.7]
V39	760.1 [700.7-907.8]	32.8 [31.2-35.7]	57.8 [53.3-73.6]	92.3 [86-108.9]	93.3 [77.8-110.8]	94.8 [82.2-136.7]	50.3 [24.3-77.1]	25.3 [24.3-26.3]
V40	102.5 [87.7-146.9]	0 [0-0]	31.4 [19.6-90.2]	90.5 [61.6-105.8]	68.1 [52.1-72.2]	41.7 [40.9-88.5]	110.2 [57.5-112.8]	0 [0-0]
V41	0 [0-0]	0 [0-0]	0 [0-0]	0 [0-0]	40.7 [26.2-44.9]	31.8 [29.7-67.2]	54.5 [26.3-57.6]	0 [0-0]

V42	23.6 [0-24.2]	35.9 [34.1-39.1]	43.2 [37-67.8]	0 [0-0]	34.1 [25-42.4]	29.2 [25.3-63.3]	45.1 [25-51]	0 [0-0]
V43	0 [0-0]	148.7 [141.3-161.8]	130.5 [117-181.1]	255.7 [203.5-265.8]	0 [0-0]	0 [0-0]	183.1 [86.4-232.4]	38.5 [32.9-44]
V44	0 [0-0]	0 [0-0]	1661 [1539.9-2465.8]	4023.2 [3315.8-4639.1]	94.8 [89.6-112]	131.8 [101-174.6]	4261.8 [2808.9-4575]	163.9 [155.7-172.1]
V45	0 [0-0]	0 [0-0]	0 [0-0]	0 [0-0]	0 [0-0]	0 [0-0]	0 [0-0]	83 [77.7-88.4]
V46	122.8 [112.6-192.1]	173.2 [135.6-184.4]	177 [157.7-267.3]	560.4 [369.7-669.4]	281.9 [252.4-366.8]	518.3 [411-878.3]	265.6 [162.8-312.1]	4000.7 [3667.8-4333.5]
V47	0 [0-0]	0 [0-0]	1186.1 [1043.9-1650.6]	879.1 [684.6-906.2]	200.8 [167.9-246]	436.5 [379.3-609]	383.7 [278.6-415.4]	0 [0-0]
V48	0 [0-0]	0 [0-0]	0 [0-0]	0 [0-0]	0 [0-0]	0 [0-0]	0 [0-0]	705.3 [633.3-777.3]
V49	0 [0-0]	0 [0-0]	0 [0-0]	0 [0-0]	0 [0-0]	0 [0-0]	0 [0-0]	860.3 [757.1-963.6]
V50	0 [0-0]	0 [0-0]	0 [0-0]	0 [0-0]	0 [0-0]	0 [0-0]	0 [0-0]	400.8 [361.3-440.2]
V51	0 [0-0]	0 [0-0]	0 [0-0]	0 [0-0]	0 [0-0]	0 [0-0]	0 [0-0]	619.2 [542-696.5]
V52	0 [0-0]	79 [75-86]	190.3 [153.5-223.5]	380.8 [243.5-415.8]	97 [65-107]	226.1 [167.9-348.7]	0 [0-0]	0 [0-0]
V53	3719.8 [2669.4-3802.8]	4234.5 [4024-4609.7]	6937 [6041.1-8556]	13178.9 [10985.5-14691]	2227.4 [2038.4-2690.4]	12372.6 [10478.4-18194.5]	1988.8 [1339.6-2041]	2194.3 [1913.1-2475.5]
V54	0 [0-0]	226.2 [215-246.2]	129.4 [119.2-188.1]	0 [0-0]	90.2 [71.3-114.6]	111.5 [78.5-223.4]	0 [0-0]	0 [0-0]
V55	0 [0-0]	216.8 [206.1-236.1]	0 [0-0]	0 [0-0]	113.1 [93.7-217.2]	97.1 [85.8-195.9]	0 [0-0]	0 [0-0]
V56	0 [0-0]	0 [0-0]	138.8 [125.6-183.6]	346.8 [133-443]	100.5 [78.9-180.4]	164.4 [152.6-324.8]	0 [0-0]	0 [0-0]
V57	65800.6 [50737.6-71692.7]	187122.9 [168168.7-267330]	100960 [90932.3-133823.1]	259080.1 [179964.9-301712.1]	133489.4 [106980.6-158759.1]	114575.2 [98904.2-188687.4]	207314.7 [130634.8-224023]	16373.1 [15959.4-16786.7]
V58	369.1 [300.9-377.3]	0 [0-0]	959.6 [802.4-1106.1]	1241.7 [918.8-1501.9]	343.1 [240-442.8]	1328.2 [1128-1668.1]	361.9 [256.7-502.5]	256.2 [230.8-281.5]
V59	341.3 [317.9-368.3]	1937.7 [1861.2-3023.6]	2798.1 [2331.5-3426]	3419.4 [2516.5-3425.3]	612.6 [527.3-655.6]	4728.6 [3832.9-6610.7]	642.9 [415.7-778.8]	1648.4 [1557.5-1739.3]
V60	0 [0-0]	0 [0-0]	0 [0-0]	0 [0-0]	0 [0-0]	0 [0-0]	0 [0-0]	2002.9 [1719.9-2285.8]
V61	0 [0-0]	0 [0-0]	1321.3 [1276-1490.7]	834.5 [680.5-1047.1]	355.2 [248.6-451.4]	1394 [951.3-1672.2]	253.8 [241.8-402.9]	0 [0-0]
V62	0 [0-0]	0 [0-0]	0 [0-0]	0 [0-0]	0 [0-0]	0 [0-0]	0 [0-0]	102.8 [60.9-144.7]
V63	0 [0-0]	0 [0-0]	0 [0-0]	0 [0-0]	0 [0-0]	0 [0-0]	0 [0-0]	2261.2 [1963.3-2559.2]
V64	0 [0-0]	837.1 [836.3-855.9]	1153.7 [1089.6-1333.8]	2761.1 [1917.6-3068.2]	302.3 [174.5-394]	2028.6 [1670.9-3342]	379.9 [257.2-446.3]	879.5 [52.7-1706.4]
V65	268 [242.4-529.4]	1054.1 [1008.9-1701.8]	1053.3 [982-1370.5]	1287.8 [711.8-1307.6]	760 [619.8-994.2]	456.7 [375.8-873]	1125.4 [452.3-2259.1]	124.7 [94.9-154.5]
V66	129.8 [69.9-493.4]	44 [41.9-47.9]	0 [0-0]	0 [0-0]	0 [0-0]	0 [0-0]	0 [0-0]	0 [0-0]
V67	329.2 [183.1-1116.4]	183.2 [166.1-183.2]	0 [0-0]	0 [0-0]	0 [0-0]	0 [0-0]	0 [0-0]	0 [0-0]
V68	0 [0-0]	0 [0-0]	0 [0-0]	0 [0-0]	0 [0-0]	0 [0-0]	0 [0-0]	778.6 [739.5-817.6]
V69	379.7 [331-399.5]	384.6 [259.3-646.6]	1001.3 [913-1176.2]	1477.1 [1289-1487.7]	242.4 [239.5-306.1]	1653.8 [1098.4-2035.6]	296.5 [163.1-301.4]	193.9 [162.6-225.3]
V70	132 [92.3-146.1]	80.3 [76.3-87.4]	139.7 [107.1-161.2]	147.9 [94.1-152.2]	122 [92.2-131.7]	191.4 [135.3-356.1]	124.9 [64.8-140.8]	0 [0-0]
V71	66 [53.2-91.5]	159.6 [138.9-256.1]	145.7 [123.6-149]	239.8 [165.7-256.5]	104.5 [96.8-125.1]	194.1 [146.9-349.4]	225.1 [110.3-231.6]	0 [0-0]
V72	194.1 [131-198.4]	451.8 [248.6-452]	0 [0-0]	479.5 [311-528.6]	0 [0-0]	632.1 [382.9-870.2]	0 [0-0]	1412.9 [1410.4-1415.3]
V73	0 [0-0]	0 [0-0]	511.7 [432.3-632.8]	518.7 [351-573.6]	176.9 [129.6-211.8]	0 [0-0]	495.8 [192.6-504.2]	46.7 [46.5-46.8]
V74	223.6 [148-228.6]	332.5 [264.7-409.4]	629.7 [527.2-728]	992 [672.8-998.7]	241.6 [182.1-265]	737.5 [555.5-1166.6]	301.5 [184-316.1]	124.3 [120-128.7]
V75	241.9 [176.8-322.1]	143 [135.9-155.7]	153.2 [122.4-160.6]	249.2 [157.8-258.7]	237.8 [154.2-260.6]	198.7 [132.2-386]	191.8 [97.4-219.9]	0 [0-0]
V76	0 [0-0]	0 [0-0]	0 [0-0]	0 [0-0]	0 [0-0]	0 [0-0]	90.9 [50.3-123.6]	0 [0-0]
V77	0 [0-0]	0 [0-0]	0 [0-0]	0 [0-0]	124.9 [86.6-132.1]	0 [0-0]	0 [0-0]	1010.8 [928-1093.7]
V78	182.1 [126.6-197.5]	1007.7 [1000-1012.1]	910.3 [836.5-1066.8]	1340.7 [933.3-1708.2]	208.2 [145.9-249.5]	1152.6 [886-2028.7]	466.5 [261.6-523.7]	92.5 [82.8-102.2]
V79	211.2 [204.8-215.9]	1085.4 [1031.4-1181.5]	1625.6 [1500.3-1852.7]	2221.4 [1582.7-2533.6]	690.3 [527.8-777.9]	806.8 [546.4-1199.6]	1134.4 [543.8-1241.2]	112.4 [108.6-116.2]
V80	2086.5 [1430.6-2133.1]	2472.1 [2349.2-2691.1]	4003.3 [3678.9-4506.6]	3401 [2012.7-3736]	1713.3 [1234-2043.8]	5576.7 [4298.7-8612.9]	1633.2 [474.6-2088.3]	0 [0-0]

[a] For variable identification (from V1 to V80) please see Table 1.

Geographical origin os samples: GB- Fontanarosa; EC- Capriglia; AP- Lacedonia; BN- Benevento; TL- Caserta (Raviscanina); MC- Ottaviano; RR. Agerola; and RRWT- Agerola (spontaneous ecotype)

Table S2. VOCs selected by Kruskal-Wallis test controlling FDR by Benjamini-Hochberg procedure (δ=0.01); p is the p-value of the Kruskal-Wallis test.

ID	P
V1	1.8E-04
V7	3.9E-04
V15	1.8E-04
V16	1.8E-04
V17	1.8E-04
V24	1.8E-04
V29	1.9E-03
V35	2.6E-03
V47	6.5E-04
V52	9.9E-04
V53	5.2E-04
V58	7.5E-04
V59	6.9E-04
V61	4.5E-04
V64	4.5E-04
V66	1.8E-04
V67	2.2E-04
V69	8.2E-04
V74	9.1E-04
V80	3.0E-03

Table S3. Spearman's correlation coefficient ρ and its p-value (p) for the VOCs selected by FDR (δ=0.05).

ID	ρ	p
V34	-0.717	1.2E-04
V5	-0.675	4.1E-04
V4	-0.670	4.7E-04
V55	-0.637	1.1E-03
V14	-0.618	1.7E-03
V22	-0.601	2.4E-03
V26	-0.589	3.1E-03
